# Diagnostic Overshadowing in Functional Neurological Disorder Leading to a Diagnosis of Acute Motor and Sensory Axonal Neuropathy: A Case Report

**DOI:** 10.3390/jcm15093501

**Published:** 2026-05-03

**Authors:** Alicia Roldan, Julieanne Shulman, Rohini Singh, Eli Dayon, Andrew Abdou, Josette Hartnett, Erika L. Trovato

**Affiliations:** 1 Glendale Memorial Hospital and Health Center, Glendale, CA 91204, USA; roldan@livevitalis.com; 2 Albert Einstein College of Medicine, Montefiore Health System, Bronx, NY 10461, USA; jushulma@montefiore.org (J.S.); aabdou@montefiore.org (A.A.); 3 The Burke Rehabilitation Hospital, White Plains, NY 10605, USA; edayon@burke.org (E.D.); johartnett@burke.org (J.H.); 4 Rehabilitation and Electrodiagnostics Physician Associates, Tampa, FL 33602, USA; drsingh@pmrdocs.com

**Keywords:** acute motor and sensory axonal neuropathy, functional neurologic disorder, case report, diagnostic overshadowing

## Abstract

**Background**: Features intersecting neurological and psychiatric disorders impose differential diagnostic challenges, especially in younger, healthy patients. Cognitive biases, such as diagnostic overshadowing, can lead to errors for patients with neurologic deficits in the presence of psychiatric comorbidities. **Methods**: This case report describes a 22-year-old female patient admitted for acute inpatient rehabilitation with an initial primary diagnosis of Functional Neurologic Disorder (FND), who subsequently underwent additional neurologic work-up following clinical and functional inconsistencies. **Results**: Physical exam findings, lack of response to therapeutic modalities, and electromyography/nerve conduction study findings led to a full neurological work-up consistent with Acute Motor and Sensory Axonal Neuropathy (AMSAN), treated with intravenous immunoglobulin. **Conclusions**: Systemic peripheral neuropathies must be addressed during differential diagnosis in suspected FND, a potential gap in current practice. This report emphasizes the essential role of physiatry and the value of an unbiased, patient-centered approach, integrating clinical knowledge and compassion to ensure accurate diagnosis and appropriate treatment.

## 1. Introduction

Despite the absence of Physical Medicine and Rehabilitation-specific epidemiologic reports, functional neurological disorder (FND) is assumed to be highly prevalent, as it has been cited to be the second most common diagnosis for new outpatient consultations in general neurology clinics [1]. FND is a notoriously challenging condition with several known subtypes. Diagnostic criteria have evolved over time, though unfortunately remain contentious. For example, FND has been categorized as a condition typified by sensorimotor disturbances inconsistent and incongruent with other known pathologies or diseases [2,3]. The International Classification of Diseases (ICD)-10 defines FND by meeting the following criteria [4]:

- One or more symptoms of altered voluntary motor or sensory function:
  - Motor symptoms (e.g., weakness, paralysis, tremors, dystonia);
  - Sensory symptoms (e.g., numbness, tingling, pain).
- Evidence of incompatibility between the symptom and recognized neurological or medical conditions:
  - Neurological examination findings that are inconsistent with the reported symptoms;
  - Absence of underlying medical conditions that could explain the symptoms;
  - The symptom or deficit is not better explained by another mental disorder;
  - The symptoms are not primarily caused by anxiety, depression, or other psychiatric conditions.

The Diagnostic and Statistical Manual, Fifth Edition, 2013 Text Revision (DSM-5 TR) [5] offers similar criteria, further emphasizing that the symptoms should not be better explained by neurologic disease, nor should the diagnosis simply be made because results from an examination are considered normal or the presence of “bizarre” symptoms. Some have argued vehemently that all current diagnostic criteria for FND are insufficient, given their reliance on the incongruence criterion, which may inadvertently promote a diagnosis of exclusion, and increase the risk of misdiagnosis [6]. Furthermore, recent perspectives have similarly contended that current diagnostic criteria for FND fail to adequately account for the possibility of underlying mechanisms for the observed symptoms [7,8]. Further noting that neurologists, who are often first-line diagnosticians, are not omniscient. That is to say, the science of neurology and understanding of brain mechanisms underlying disorders have advanced, with diagnoses hinging on incongruency, ignoring these facts [3,8].

Examination of internal consistency, such as when a symptom is present in some contexts but not others, may increase diagnostic confidence for a FND diagnosis. Additionally, identifying positive clinical signs considered characteristic of FND is proposed, such as shoulder tap with amplified movements and dissociated movement patterns, such as running movements of arms in the absence of corresponding leg movements [9,10]. Finally, diagnostic accuracy can be enhanced by assessing comorbid symptomology; overlapping features in the setting of the absence of expected accompanying signs are important. The example of freezing gait is highlighted in the context of Parkinsonism, whereas freezing of gait in isolation, without other characteristic Parkinsonism symptoms, would raise suspicion of a functional disorder.

In addition to a comprehensive neurologic examination, diagnostic evaluations for neurological conditions may include: imaging of brain and spine, electroencephalogram (EEG), lumbar puncture (LP), evaluation of the peripheral nervous system using electrodiagnostic modalities, and neuropsychological assessment (NPA) [11,12]. However, in routine clinical practice, patients with suspected FND, particularly those with a history of psychiatric conditions, do not consistently undergo neuroimaging or peripheral nervous system evaluation as part of the standard diagnostic process [10].

Previously understood as a conversion disorder, a hallmark of FND is the high degree of variability in symptomatology, complicating diagnosis and treatment [2]. Some clinicians have questioned the appropriateness of inpatient rehabilitation for treating this condition [13]. However, a series of recommendations and reviews published from 2015 to 2023 advocate for the primary role of interdisciplinary rehabilitation [13,14,15] and outline FND-specific strategies [14,16,17,18,19], including guidelines for physical therapists [19], occupational therapists [13], and speech–language pathologists [20], with a primary focus on the motor and gait impairment subtype.

Historically, patients diagnosed with FND were primarily managed by mental health professionals alone; FND has grown pervasive enough in neurology practices within the last decade that some describe it as reaching epidemic levels [21]. Similarly, among the most common diagnoses seen in neurology clinics, FND patient presentations have been reported to be second only to headaches [22,23,24]. Some have suspected FND is the actual cause of as many as 10% of inpatient admissions and 8% of suspected stroke hospital presentations [25,26], with a recent study indicating that approximately one in five patients hospitalized for acute motor FND require discharge to inpatient rehabilitation [27].

It is well known that there is a high incidence of mood disorders that co-occur with neurologic conditions, such as stroke, Parkinson’s disease, and Multiple Sclerosis [28,29,30]. Similarly, the phenotypic heterogeneity of FND and its intersection with both psychiatric and neurologic comorbidities are cited to increase the risk of missed or misdiagnoses [31,32,33], highlighting the relevant concept of diagnostic overshadowing. Diagnostic overshadowing first appeared in the literature in the early 1980s [34], referring to a type of cognitive bias in healthcare that misattributes symptoms of an undiagnosed physical illness to an already diagnosed disorder, disability, or comorbid psychiatric condition [35].

Recent literature has revealed a misdiagnosis rate of approximately 4% for FND in the outpatient setting [36,37] and about 11% in the inpatient setting [13,38]. Misdiagnosis is more common in patients with gait and movement disorders, as well as psychiatric histories [37], and approximately 20% of FND cases involve comorbid neurological conditions [8,31,39]. With a primary role in the rehabilitation of motor FND patients, a deeper understanding of this disorder is vital for the physiatric community, particularly in terms of diagnostics and rehabilitation strategies. Physiatrists should therefore consider ordering a work-up for functional neurologic disorders when clinical features point toward suspicion of primary or comorbid FND.

Furthermore, neurological conditions and physical injuries—both central to the rehabilitation medicine population—are well-recognized precursors to FND [11,14]. Thus, the purpose of this report is to illustrate the potential for diagnostic overshadowing in patients with suspected FND, emphasizing the importance of continued diagnostic evaluation when clinical features extend beyond rule-in signs, and to highlight the essential role of physiatrists in identifying coexisting neurologic pathology.

## 2. Case Presentation

This case report describes a twenty-two-year-old single cisgender Caucasian female with a history of International Classification of Diseases (ICD) 11 [40] diagnoses of Major Depressive Disorder, recurrent with psychotic features (ICD-11-CM: 6A71.3), Obsessive–Compulsive Disorder (OCD) (ICD-11 Code: 6B20), and Post-Traumatic Stress Disorder (PTSD) (ICD-11 Code: 6B40), with one remote suicide attempt and two lifetime psychiatric hospitalizations. The patient had a history of non-compliance with her psychopharmacology and had been disconnected from treatment for some time.

Upon hearing that her daughter was not well, the patient’s mother arrived from New York (NY), finding that her daughter had become bed-bound with severe bilateral lower extremity weakness (BLE), inability to complete activities of daily living (ADLs), and symptoms consistent with neuropathic pain. Reports also suggest functional decline along with bouts of gross incontinence. At that time, the patient was taken to the Emergency Department (ED) and diagnosed with a urinary tract infection (UTI). She was subsequently discharged home and transitioned to the care of her mother in another state. Following multiple falls in the setting of worsened BLE weakness accompanied by bowel and/or bladder incontinence, she was brought back to the ED. Physical examination was notable for significant weakness in the proximal bilateral lower extremities and impaired balance.

The patient’s clinical presentation was inconsistent, particularly with respect to her functional status. For example, the patient was documented as standing independently; however, she would then lower herself to the floor, stating she could not stand. The treatment team subsequently determined that the patient’s condition was due to functional weakness. Simultaneously, the treatment team noted worsening depression and suicidal ideation, and the patient was then discharged to a psychiatric hospital where she was diagnosed with FND. During her two-month psychiatric hospitalization, her functional status worsened, necessitating admission to an acute inpatient rehabilitation facility (IRF).

### Assessment

Once admitted to the IRF, the patient reported diarrhea, nausea, poor appetite, fatigue, dysuria, and tingling in her hands and feet. She also complained of knee pain that she stated was due to a patellar fracture from a fall onto her knees weeks prior. Physical exam revealed hypophonia of uncertain etiology and antigravity weakness of the distal bilateral upper extremity (BUE) with flexed positioning of the wrists. Additionally, there was weakness of the bilateral knees and ankles, tight Achilles tendons, areflexia sparing the bilateral brachioradialis only, and inconsistent bowel and/or bladder incontinence. Proximal bilateral shoulder and hip flexor strength could resist moderate examiner power. Psychiatrically, the patient presented with symptoms consistent with depressed mood with congruent, flat affect and behavioral withdrawal. Anxiety was noted to be a salient feature of her presentation as well. Admission labs were unremarkable, including a urinalysis, as was knee x-ray imaging. Medication reconciliation at the time of IRF admission included aripiprazole, fluoxetine, and modafinil to address mood symptoms.

Multidisciplinary treatment was initiated, including neuropsychology for cognitive behavioral therapy (CBT), dietary/nutrition consultations given poor oral intake, and physical, occupational, and speech therapy. Psychiatry was consulted, and medications were adjusted, including discontinuation of modafinil, cross-titration of fluoxetine to venlafaxine, initiation of melatonin for sleep, and alprazolam for anxiety. While features were consistent with FND, several were more typical of other known neurologic disorders—for example, the patient’s physical exam was remarkable for symmetrical BLE weakness as well as diffuse areflexia. Given the patient’s lack of functional progression after several days, electrical stimulation was trialed on the BLE. Physical therapy reported that no response was elicited, prompting immediate orders for an electromyography (EMG) and a nerve conduction study (NCS).

## 3. EMG and NCS Findings

Sensory NCS at multiple stimulation sites showed either no responses or low amplitudes, which is consistent with severe axonal damage (Table 1).

Altogether, the low and absent responses in the bilateral median, ulnar, radial, superficial peroneal, and sural sensory nerves indicated widespread sensory axonal involvement. Motor NCS revealed significantly reduced amplitudes in right median and ulnar motor nerves (the left side was not tested), with no responses in the bilateral peroneal and tibial motor nerves, further underscoring severe axonal degeneration (Table 2).

Finally, needle EMG studies showed increased spontaneous activity and partial or absent volitional activity interference patterns in various muscles, which are all significant findings indicative of active denervation (Table 3).

### Neurologic Diagnosis Obscured by FND and Psychiatric Comorbidities

Following the EMG, the patient was emergently transferred to the acute hospital setting. Extensive neurologic workup, including CT head without contrast, MRI of the brain and cervical spine, with and without contract, LP, and paraneoplastic evaluation, all resulted in negative findings (Supplementary Material File). Urine toxicology screen was negative. Given EMG and NCS findings indicating extensive axonal damage in both motor and sensory nerves characteristic of presumed Acute Motor and Sensory Axonal Neuropathy (AMSAN), the patient was treated with a course of intravenous immunoglobulin (IVIG) [41] without any adverse events occurring during or post-infusion.

Following IVIG treatment, the patient returned to the IRF in early February, with mild improvement in proximal bilateral upper extremity strength. The patient reported feeling hopeful, less anxious, and no longer depressed. She felt her anxiety was well controlled and was sleeping better, hoping to be discharged home under her mother’s care once the needed home services and supports were installed. She was able to actively discuss her goals with the multidisciplinary teams and had increased participation in therapy overall. The patient’s medications were further titrated to only include aripiprazole, venlafaxine, and alprazolam.

Prior to the discovery of AMSAN, the patient demonstrated minimal functional gain, and discharge to a subacute rehabilitation setting was anticipated. After receiving IVIG treatment, her functional recovery in the inpatient rehabilitation setting enabled a home discharge three weeks later. Discharge planning included installing a Hoyer lift and a ramp, and 24 h, 7-days-per-week caregiver support for safety and assistance with ADLs.

## 4. Discussion

### 4.1. Dual Diagnosis: FND and AMSAN

Physiatrists have a significant role in the treatment pathway of FND, given their skills in rehabilitation and neurologic contexts. Similarly, they have a responsibility to engage in critical reflection of atypically presenting symptoms to prevent potentially harmful diagnostic bias. For this patient, strength testing demonstrated only mild improvement, specifically in gravity-eliminated bilateral knee extension, trace movement in bilateral hamstrings, and 4/5 strength in bilateral hip flexion. Strength had been largely preserved at the shoulders with mild weakness at the elbows throughout her hospitalization. Despite these functional improvements, she remained at the Hoyer lift level for transfers, both due to body habitus and psychiatric limitations.

While AMSAN accounted for many of the patient’s symptoms, certain clinical features remained unexplained during the differential diagnosis between FND and AMSAN. Notably, hypophonia, though improved, persisted at discharge and, in isolation, is difficult to attribute to AMSAN. The inconsistent course of the patient’s sensory symptomology is similarly uncharacteristic of AMSAN [11]. Speech problems such as aphonia, dysphonia, stuttering, dysarthria, mutism, and foreign accent syndrome are well documented in FND; however, these manifestations are not specific and may also occur in structural and other neurological disorders [10,24]. Similarly, AMSAN literature indicates that bowel and/or bladder incontinence is not correlated with this diagnosis [42]. In fact, bowel and/or bladder involvement at onset or that persists is cited as an atypical feature in AMSAN that should prompt a provider to consider a different diagnosis [42,43]. Additional atypical presentations that should raise doubt concerning demyelinating disorders include marked persistence of asymmetric features and hyperreflexia or normal reflexes.

Organic pathologic disease that preferentially affects the strongest muscle groups—flexors in the UE and extensors in the LE—is unusual [24]. The presentation and progression of the patient’s weakness and bilateral, symmetric, distal extremity tingling were consistent with AMSAN. Sensory deficits in FND patients usually lack a clear dermatomal or peripheral nerve distribution and may vary in severity and location with time [11]. Additionally, in functional diagnoses, limb weakness is most often unilateral and diffuse, affecting flexors and extensors equally. This patient’s reported weakness and tingling sensations were largely symmetric and reflected the ascending pattern of AMSAN, more severely affecting the lower than the upper extremity. Her return of function additionally proceeded proximally to distally, as would be expected. Importantly, the diagnosis of AMSAN did not eliminate the diagnosis of FND, with both diagnoses coexisting, highlighting the frequent concomitance of FND symptomatology and other neurologic illnesses (Table 4).

### 4.2. Diagnostic Overshadowing and Challenges

By the time the patient was admitted to the IRF, the FND diagnosis gained momentum, leading to a cognitive bias that shaped the lens through which the features of the patient’s illness were initially seen. Variability in the patient’s examined and observed strength, effort, and capacity supported the FND diagnosis. However, the symmetry and severity of bilateral lower extremity weakness with tight heel cords was suspicious. Highlighting the importance of interdisciplinary teams and communication in rehabilitation, it was not until the PT evaluation with electrical stimulation yielded objective evidence of a potential additional etiology to explain the patient’s constellation of symptoms that further work-up was initiated, corresponding to previous reports on the effectiveness of physiotherapy for patients with FND [44].

Diagnostic overshadowing has been described as a departure from clinically accepted principles of developing a differential diagnosis [45]. In the case of FND, a complex disorder with nuanced features that intersect with psychiatry and neurology, a thorough differential diagnosis considering all clinical features, natural history, and response to treatment is imperative. This case exemplifies the potential for the cognitive bias of diagnostic overshadowing to further complicate diagnostic assessment. In this case, both the challenges that can arise in accurately diagnosing neurologic deficits in the presence of psychiatric comorbidities, and the challenge of diagnosing FND in the setting of concomitant neurologic pathology [46].

The patient’s medical care was distributed over two states, with treatment settings including acute medical and inpatient psychiatric settings, beginning with a presentation of decompensated major depression, anxiety, and UTI, later followed by colitis. The patient’s psychiatric condition became the lens through which all other symptoms were seen. Similar to previous reports [47], one can perhaps understand how the patient’s psychiatric symptomatology could become the clinical focus.

It is typical for patients with multiple medical comorbidities to move through multiple levels or tiers of care, and treatment across different settings can further contribute to diagnostic overshadowing. As has been previously noted in FND literature, recognizing clinical features that are inconsistent with known neurologic disorders is an important diagnostic criterion that may require expertise not limited to psychiatry but inclusive of the diagnostic impressions of an experienced neurologist [48]. Different treatment settings may further contribute to diagnostic overshadowing and misdiagnoses if appropriate specialists or infrastructure are lacking [7].

Once an initial diagnosis is made, it can sometimes take hold, unconsciously reducing a physician’s ability to consider other alternatives [49]. The consequences can involve compromised or delayed diagnosis and treatment, inadequate or unsafe patient care, and inequities in care [50]. Comorbid psychiatric diagnoses can raise this risk. In fact, diagnostic overshadowing is documented extensively in the medical literature, particularly with those most vulnerable, including patients with intellectual disabilities, physical disabilities, neurologic deficits, psychiatric comorbidities, substance abuse, obesity, with those who possess low health literacy, and for patients who identify with Lesbian, Gay, Bisexual, Transgender, and Queer (LGBTQ) communities [45]. The detriment of the cognitive bias inherent in diagnostic overshadowing cannot be underestimated, particularly in the case of reversible conditions where a delayed diagnosis can be fatal or permanently impairing.

### 4.3. Rehabilitation Strategies for Motor FND Differ from Traditional Rehabilitation Approaches

Although the patient’s motor and sensory deficits are most significantly due to AMSAN, the diagnosis and clinical expression of concomitant FND warrant a rehabilitation approach that follows FND-specific recommendations, given the neurobiological understandings of this condition. Several neuroimaging studies of FND patients have begun to reveal alterations in multiple brain networks, including within the limbic system and salience networks, potentially affecting the individual’s perception of voluntary control and agency over physical movements, as well as difficulty integrating sensory information and emotional processing [11]. While currently there is no gold standard of treatment, emerging literature suggests some benefit of physical rehabilitation in combination with CBT in the management of motor FND patients [14].

Unlike rehabilitation for traditional movement disorders, rehabilitation techniques for functional disorders can involve focusing on basic, functional, and automatic movement patterns rather than specific impairments. For example, some patients have exhibited enhanced ambulatory capacity on a treadmill or improved movement patterns through automatic motions of dance [24,51]. Distraction away from self and the targeted movement patterns often reduces symptom severity and can trigger normal movement patterns, allowing for motor retraining in FND patients. This shift in attention and focus away from the deficit and matters of agency is contrary to the techniques of traditional physical therapy. Some examples of distraction techniques used include snapping, listening to music, and counting backwards [51]. One randomized feasibility study of 60 patients with FND demonstrated that compared to standard therapy, one week of intensive therapy (at least 5 sessions/week) led to a 72% improvement maintained at six months, with only a 28% improvement found for patients receiving standard care [52]. Occupational therapists are acknowledged as particularly well-suited for the treatment of FND patients, as they have a skillset focused on day-to-day movement patterns and activities rather than rehabilitation for physical impairments alone [24].

### 4.4. Helpful Considerations for the Physiatrist

Diagnosing FND has historically fallen under the auspices of neurology and psychiatry. However, as part of the primary treatment team, it is incumbent upon physiatrists to question the presence of conflicting clinical signs that may indicate a missed or misdiagnosis. Appropriate diagnostic workup should not be prematurely discontinued when clinical features extend beyond the signs used to support a FND diagnosis [48]. In a review of FND for the general practitioner, physicians are encouraged to always ask themselves whether it is possible that a patient with a clear FND diagnosis may have FND and another condition [24]. Physiatrists must balance the importance of avoiding unnecessary testing and iatrogenic harm with the knowledge that a significant percentage of FND patients, particularly those with chronic disease, may have another underlying clinical pathology [7]. For instance, 20% of individuals with the psychogenic non-epileptic seizure subtype of FND have comorbid epileptic seizures [53], and FND is known to coexist in a subset of individuals with Parkinson’s Disease [54].

Finally, with respect to qualifying patient progress in rehabilitation, there is emerging evidence for at least short-term functional improvements with inpatient rehabilitation, especially for patients newly diagnosed with FND [13]. Significant improvements were observed in patients receiving one week of at least five FND-specific rehabilitation sessions, with improvements maintained at six months follow-up after only one week of at least five physiotherapy sessions [52]. The phenotypic heterogeneity of this patient population can make it difficult to determine which metrics to focus on for measuring outcomes. For instance, research on motor FND, including a randomized controlled trial [55], found that for these patients, pain, anxiety, cognitive symptoms, fatigue, and depression correlated more strongly with their perception of quality of life than the symptoms of their movement disorder. Considering that meaningful outcome measures may vary significantly from the perspective of the physician versus the patient, it is important to consult the patient regarding which symptoms are of most importance when selecting outcomes on which to measure the patient’s progress [2,56].

## 5. Conclusions

With a well-established primary role to serve in the treatment of FND and increasing recognition of the prevalence in our patient population, rehabilitation physicians must ensure a comprehensive understanding of the diagnostics and rehabilitation strategies that are unique to this diagnosis. Presenting disabilities at the intersection between psychiatry and neurology, FND patients are vulnerable to health inequities created by physicians through diagnostic overshadowing. Improper or delayed diagnosis of FND and/or underlying neurologic comorbidities can have significant consequences as severe as the premature death of patients. The potential medical errors and inadequate care associated with diagnostic overshadowing further add increasing costs to an already financially strained healthcare system, in which expenditures attributable to FND are conservatively measured at more than $1.2 billion dollars annually. As the prevalence and financial burden of this patient population grows, physiatrists and other rehabilitation professionals will increasingly find themselves called to contribute to the body of research needed to establish gold standards of rehabilitation care for patients with FND. Given the musculoskeletal and neurologic expertise of physiatrists and the interdisciplinary teamwork of IRFs, few specialties are better poised to meet the unique challenges posed by this multifaceted diagnosis and sometimes complex patient population.

## Figures and Tables

**Table 1:** Sensory nerve conduction study (SNCS) findings.

Nerve (Recording Site)	Stimulus Site	NR	Onset (ms)	Peak (ms)	Normal Peak (ms)	O–P Amplitude (µV)	Normal O–P Amp	Distance (cm)	Velocity (m/s)	Normal Velocity (m/s)
Left Median (D2)	Wrist → 2nd digit		5.5	6.0 *	<4	2.4 *	>11	14.0	25 *	>40
Right Median (D2)	Wrist → 2nd digit	NR *			<4		>11	14.0		>40
Left Radial	Radial forearm → 1st web space	NR *			<2.8		>7	0.0		>40
Right Radial	Radial forearm → 1st web space	NR *			<2.8		>7	0.0		>40
Left Superficial Peroneal	Lateral calf → ant. lateral ankle	NR *			<4.2		>4	14.0		>40
Right Superficial Peroneal	Lateral calf → ant. lateral ankle	NR *			<4.2		>4	14.0		>40
Left Sural	Posterior calf → lateral malleolus	NR *			<4		>4.5	14.0		>40
Right Sural	Posterior calf → lateral malleolus	NR *			<4		>4.5	14.0		>40
Left Ulnar (D5)	Wrist → 5th digit	NR *			<4		>10	14.0		>40
Right Ulnar (D5)	Wrist → 5th digit	NR *			<4		>10	14.0		>40

Abbreviations: NR, no response; ms, milliseconds; µV, microvolts; O–P, onset-to-peak. * = Abnormal value.

**Table 2:** Motor nerve conduction study (MNCS) findings.

Nerve (Muscle)	SS	NR	Onset (ms)	Normal Onset (ms)	O–P Amp (mV)	Normal O–P Amp	Distance (cm)	Velocity (m/s)	Normal Velocity (m/s)
Right Median (APB)	Wrist	-	3.0	<4.5	0.3 *	>4.1	22.0	51	>49
	Elbow	-	7.3	-	0.2	-	-	-	-
Left Peroneal (EDB)	Ankle	NR *	-	<6.5	-	>1.3	-	-	>38
Right Peroneal (EDB)	Ankle	NR *	-	<6.5	-	>1.3	-	-	>38
Left Tibial (AHB)	Ankle	-	9.7 *	<6.1	0.2 *	>4.4	36.0	39	>39
Right Tibial (AHB)	Ankle	NR *	-	<6.1	-	>4.4	-	-	>39
Right Ulnar (ADM)	Wrist	-	4.2 *	<3.7	0.7 *	>7.9	17.0	52	>52

Abbreviations: APB, abductor pollicis brevis; EDB, extensor digitorum brevis; AHB, abductor hallucis brevis; ADM, abductor digiti minimi; NR, no response; ms, milliseconds; mV, millivolts, Stimulus Site, SS. * = Abnormal value.

**Table 3:** Needle electromyography findings.

Side	Muscle	Nerve	Root	IA	Fibs	PSWs	Amp	Dur	Polyph	Recru	IP
Right	*Extensor Hallucis Longus*	Deep Peroneal	L5–S1	Normal	0	0	Normal	Normal	Normal	Normal	Complete (4+)
Right	*Anterior Tibialis*	Deep Peroneal	L4–5	Normal	1+ *	3+ *	Normal	Normal	Normal	Normal	No Volitional Activity (0) *
Right	*Medial Gastrocnemius*	Tibial	S1–2	Normal	1+ *	2+ *	Normal	Normal	Normal	Normal	Partial (2+) *
Right	*Vastus Medialis*	Femoral	L2–4	Normal	0	1+ *	Normal	Normal	Normal	Normal	No Volitional Activity (0) *
Left	*Extensor Hallucis Longus*	Deep Peroneal	L5–S1	Normal	0	0	Normal	Normal	Normal	Normal	Complete (4+)
Left	*Anterior Tibialis*	Deep Peroneal	L4–5	Normal	2+ *	3+ *	Normal	Normal	Normal	Normal	No Volitional Activity (0) *
Left	*Medial Gastrocnemius*	Tibial	S1–2	Normal	1+ *	3+ *	Normal	Normal	Normal	Normal	Partial (2+) *
Left	*Vastus Medialis*	Femoral	L2–4	Normal	0	2+ *	Normal	Normal	Normal	Normal	Single Motor Unit (1+) *
Bilateral	*Lumbar Paraspinals (L3–S1)*	Rami	L3–S1	Normal	0	0	Normal	Normal	Normal	–	–
Bilateral	*Flexor Carpi Radialis*	Median	C6–7	Normal	0	2+ *	Normal	Normal	Normal	Normal	Partial (2+) *

Abbreviations: Dur, Duration, Fibs, fibrillations; Polyphasia, Polyph, PSWs, positive sharp waves; Insertional Activity, IA; Interference Pattern, Ips, Recruitment, Recru. * = Abnormal value.

**Table 4:** Comparison of clinical features in FND and AMSAN.

Symptom	FND	AMSAN
Bowel and/or bladder incontinence	Yes	No
Hyphonia	Yes	No
Tingling in bilateral hands and feet	No	Yes
Bilateral lower-extremity plegia/paresis	No	Yes
Tight heel cords	No	Yes
Weakness	Yes	Yes
Hyporeflexia	No	Yes
Degree of disability	Yes	–

## Data Availability

All data regarding this case report are presented in full; additional/supporting data were not collected, nor are they available on request.

## Supplementary Materials

The following supporting information can be downloaded at https://www.mdpi.com/article/10.3390/jcm15093501/s1.

## References

[1] Leochico C.F.D., Mitchell S.B., Levitt S.E., Tam A., Guo M. (2024). Leveraging Physical and Rehabilitation Medicine in the Interdisciplinary Care of Persons with Functional Neurological Disorder. J. Int. Soc. Phys. Rehabil. Med..

[2] Nicholson T.R., Carson A., Edwards M.J., Goldstein L.H., Hallett M., Mildon B., Nielsen G., Nicholson C., Perez D.L., Pick S. (2020). Outcome Measures for Functional Neurological Disorder: A Review of the Theoretical Complexities. J. Neuropsychiatry Clin. Neurosci..

[3] Stone J. (2024). Incongruence in FND: Time for Retirement. Pract. Neurol..

[4] World Health Organization (2026). ICD-10-CM Diagnosis Code F44.4: Conversion Disorder with Motor Symptom or Deficit. https://icd.who.int/browse10/2019/en#F44.

[5] American Psychiatric Association (2022). Diagnostic and Statistical Manual of Mental Disorders: Fifth Edition Text Revision DSM-5-TR^TM^.

[6] Daum C., Gheorghita F., Spatola M., Stojanova V., Medlin F., Vingerhoets F., Berney A., Gholam-Rezaee M., Maccaferri G.E., Hubschmid M. (2015). Interobserver Agreement and Validity of Bedside “positive Signs” for Functional Weakness, Sensory and Gait Disorders in Conversion Disorder: A Pilot Study. J. Neurol. Neurosurg. Psychiatry.

[7] Van Der Feltz-Cornelis C.M., Allen S.F., Van Eck Van Der Sluijs J.F. (2020). Misdiagnosis of an Underlying Medical Condition as Conversion Disorder/Functional Neurological Disorder (CD/FND) Still Occurs. Gen. Hosp. Psychiatry.

[8] Stone J., Carson A., Duncan R., Roberts R., Coleman R., Warlow C., Murray G., Pelosi A., Cavanagh J., Matthews K. (2012). Which Neurological Diseases Are Most Likely to Be Associated with “Symptoms Unexplained by Organic Disease”. J. Neurol..

[9] Edwards M.J., Yogarajah M., Stone J. (2023). Why Functional Neurological Disorder Is Not Feigning or Malingering. Nat. Rev. Neurol..

[10] Espay A.J., Aybek S., Carson A., Edwards M.J., Goldstein L.H., Hallett M., LaFaver K., LaFrance W.C., Lang A.E., Nicholson T. (2018). Current Concepts in Diagnosis and Treatment of Functional Neurological Disorders. JAMA Neurol..

[11] Mavroudis I., Kazis D., Kamal F.Z., Gurzu I.-L., Ciobica A., Pădurariu M., Novac B., Iordache A. (2024). Understanding Functional Neurological Disorder: Recent Insights and Diagnostic Challenges. Int. J. Mol. Sci..

[12] Gilmour G.S., Nielsen G., Teodoro T. (2020). Management of Functional Neurological Disorder. J. Neurol..

[13] Polich G., Zalanowski S., Lewis J.M., Sugarman S., Christopulos K., Hebb C., Perez D.L., Baslet G., Shah P., Herman S. (2023). Inpatient Rehabilitation for Acute Presentations of Motor Functional Neurological Disorder (FND): A Retrospective Cohort Study. Am. J. Phys. Med. Rehabil..

[14] Kelmanson A.N., Kalichman L., Treger I. (2023). Physical Rehabilitation of Motor Functional Neurological Disorders: A Narrative Review. Int. J. Environ. Res. Public Health.

[15] Stone J., Milburn-McNulty P. (2025). Reclaiming Functional Neurological Disorder for Rehabilitation Medicine. Adv. Rehabil. Sci. Pract..

[16] Jimenez X.F., Aboussouan A., Johnson J. (2019). Functional Neurological Disorder Responds Favorably to Interdisciplinary Rehabilitation Models. Psychosomatics.

[17] McKee K., Glass S., Adams C., Stephen C.D., King F., Parlman K., Perez D.L., Kontos N. (2018). The Inpatient Assessment and Management of Motor Functional Neurological Disorders: An Interdisciplinary Perspective. Psychosomatics.

[18] Jordbru A., Smedstad L., KlungsÃ¸yr O., Martinsen E. (2014). Psychogenic Gait Disorder: A Randomized Controlled Trial of Physical Rehabilitation with One-Year Follow-Up. J. Rehabil. Med..

[19] Czarnecki K., Thompson J.M., Seime R., Geda Y.E., Duffy J.R., Ahlskog J.E. (2012). Functional Movement Disorders: Successful Treatment with a Physical Therapy Rehabilitation Protocol. Park. Relat. Disord..

[20] Baker J., Barnett C., Cavalli L., Dietrich M., Dixon L., Duffy J.R., Elias A., Fraser D.E., Freeburn J.L., Gregory C. (2021). Management of Functional Communication, Swallowing, Cough and Related Disorders: Consensus Recommendations for Speech and Language Therapy. J. Neurol. Neurosurg. Psychiatry.

[21] Williams S., Southall C., Haley S., Ba Dhafari T., Kemp S., Relton S.D., Alty J.E., Johnson O., Graham C.D., Maguire M. (2022). To the Emergency Room and Back Again: Circular Healthcare Pathways for Acute Functional Neurological Disorders. J. Neurol. Sci..

[22] Stone J., Carson A., Duncan R., Roberts R., Warlow C., Hibberd C., Coleman R., Cull R., Murray G., Pelosi A. (2010). Who Is Referred to Neurology Clinics?—The Diagnoses Made in 3781 New Patients. Clin. Neurol. Neurosurg..

[23] Carson A., Lehn A. (2016). Epidemiology. Handbook of Clinical Neurology.

[24] Bennett K., Diamond C., Hoeritzauer I., Gardiner P., McWhirter L., Carson A., Stone J. (2021). A Practical Review of Functional Neurological Disorder (FND) for the General Physician. Clin. Med..

[25] Gargalas S., Weeks R., Khan-Bourne N., Shotbolt P., Simblett S., Ashraf L., Doyle C., Bancroft V., David A.S. (2017). Incidence and Outcome of Functional Stroke Mimics Admitted to a Hyperacute Stroke Unit. J. Neurol. Neurosurg. Psychiatry.

[26] Wilkins S.S., Bourke P., Salam A., Akhtar N., D’Souza A., Kamran S., Bhutta Z., Shuaib A. (2018). Functional Stroke Mimics: Incidence and Characteristics at a Primary Stroke Center in the Middle East. Psychosom. Med..

[27] Stephen C.D., Fung V., Lungu C.I., Espay A.J. (2021). Assessment of Emergency Department and Inpatient Use and Costs in Adult and Pediatric Functional Neurological Disorders. JAMA Neurol..

[28] Cong S., Xiang C., Zhang S., Zhang T., Wang H., Cong S. (2022). Prevalence and Clinical Aspects of Depression in Parkinson’s Disease: A Systematic Review and Meta-analysis of 129 Studies. Neurosci. Biobehav. Rev..

[29] Pagonabarraga J., Álamo C., Castellanos M., Díaz S., Manzano S. (2023). Depression in Major Neurodegenerative Diseases and Strokes: A Critical Review of Similarities and Differences among Neurological Disorders. Brain Sci..

[30] Kwon S., Han K.-D., Jung J.H., Cho E.B., Chung Y.H., Park J., Choi H.L., Jeon H.J., Shin D.W., Min J.-H. (2024). Risk of Depression and Anxiety in Multiple Sclerosis and Neuromyelitis Optica Spectrum Disorder: A Nationwide Cohort Study in South Korea. Mult. Scler. J..

[31] Finkelstein S.A., Popkirov S. (2023). Functional Neurological Disorder: Diagnostic Pitfalls and Differential Diagnostic Considerations. Neurol. Clin..

[32] Mavroudis I., Franekova K., Petridis F., Ciobîca A., Gabriel D., Anton E., Ilea C., Papagiannopoulos S., Kazis D. (2025). Comorbidities Across Functional Neurological Disorder Subtypes: A Comprehensive Narrative Synthesis. Life.

[33] Garizio J.A., Nazir H. (2023). A Great Imitator: Functional Neurologic Disorder as a Cause of Stroke Mimic. CHEST.

[34] Reiss S., Levitan G.W., Szyszko J. (1982). Emotional Disturbance and Mental Retardation: Diagnostic Overshadowing. Am. J. Ment. Defic..

[35] Jones S., Howard L., Thornicroft G. (2008). “Diagnostic Overshadowing”: Worse Physical Health Care for People with Mental Illness. Acta Psychiatr. Scand..

[36] Gelauff J.M., Carson A., Ludwig L., Tijssen M.A.J., Stone J. (2019). The Prognosis of Functional Limb Weakness: A 14-Year Case-Control Study. Brain.

[37] Stone J., Smyth R., Carson A., Lewis S., Prescott R., Warlow C., Sharpe M. (2005). Systematic Review of Misdiagnosis of Conversion Symptoms and “Hysteria”. BMJ.

[38] Teasell R.W., Shapiro A.P. (2002). Misdiagnosis of Conversion Disorders. Am. J. Phys. Med. Rehabil..

[39] Tinazzi M., Geroin C., Erro R., Marcuzzo E., Cuoco S., Ceravolo R., Mazzucchi S., Pilotto A., Padovani A., Romito L.M. (2021). Functional Motor Disorders Associated with Other Neurological Diseases: Beyond the Boundaries of “Organic” Neurology. Eur. J. Neurol..

[40] World Health Organization (2019). International Classification of Diseases, Eleventh Revision (ICD-11).

[41] Bril V., Katzberg H.D. (2014). Acquired Immune Axonal Neuropathies. Continuum.

[42] Van Den Berg B., Walgaard C., Drenthen J., Fokke C., Jacobs B.C., Van Doorn P.A. (2014). Guillain–Barré Syndrome: Pathogenesis, Diagnosis, Treatment and Prognosis. Nat. Rev. Neurol..

[43] Nguyen T.P., Taylor R.S. (2023). Guillain-Barre Syndrome.

[44] Nielsen G., Stone J., Edwards M.J. (2013). Physiotherapy for Functional (Psychogenic) Motor Symptoms: A Systematic Review. J. Psychosom. Res..

[45] Sentinel Event Alert 65: Diagnostic Overshadowing Among Groups Experiencing Health Disparities. https://www.jointcommission.org/resources/sentinel-event/sentinel-event-alert-newsletters/sentinel-event-alert-65-diagnostic-overshadowing-among-groups-experiencing-health-disparities/.

[46] Mavroudis I., Petrides F., Franekova K., Ionescu C., Ciobica A., Kazis D. (2025). Treatment of Functional Neurological Disorder: An Umbrella Review of Systematic Reviews and Meta-Analyses. J. Neurol..

[47] Geiss M., Chamberlain J., Weaver T., McCormick C., Raufer A., Scoggins L., Petersen L., Davis S., Edmonson D. (2018). Diagnostic Overshadowing of the Psychiatric Population in the Emergency Department: Physiological Factors Identified for an Early Warning System. J. Am. Psychiatr. Nurses Assoc..

[48] Perez D.L., Hunt A., Sharma N., Flaherty A., Caplan D., Schmahmann J.D. (2020). Cautionary Notes on Diagnosing Functional Neurologic Disorder as a Neurologist-in-Training. Neurol. Clin. Pract..

[49] The Joint Commission Cognitive Biases in Health Care. https://www.ashrm.org/system/files/media/file/2024/02/Patient-Safety-Tip-Sheet-Cognitive-Biases-in-Health-Care.pdf.

[50] National Council on Disability Framework (2022). Health Equity Framework for People with Disabilities. https://www.ncd.gov/assets/uploads/reports/2022/ncd_health_equity_framework.pdf.

[51] Nielsen G., Stone J., Matthews A. (2015). Physiotherapy for Functional Motor Disorders: A Consensus Recommendation. J. Neurol. Neurosurg. Psychiatry.

[52] Nielsen G., Buszewicz M., Stevenson F., Hunter R., Holt K., Dudziec M., Ricciardi L., Marsden J., Joyce E., Edwards M. (2017). Randomised Feasibility Study of Physiotherapy for Patients with Functional Motor Symptoms. J. Neurol. Neurosurg. Psychiatry.

[53] Kutlubaev M.A., Xu Y., Hackett M.L., Stone J. (2018). Dual Diagnosis of Epilepsy and Psychogenic Nonepileptic Seizures: Systematic Review and Meta-Analysis of Frequency, Correlates, and Outcomes. Epilepsy Behav..

[54] Wissel B.D., Dwivedi A.K., Merola A., Chin D., Jacob C., Duker A.P., Vaughan J.E., Lovera L., LaFaver K., Levy A. (2018). Functional Neurological Disorders in Parkinson Disease. J. Neurol. Neurosurg. Psychiatry.

[55] Gelauff J.M., Kingma E.M., Kalkman J.S., Bezemer R., Van Engelen B.G.M., Stone J., Tijssen M.A.J., Rosmalen J.G.M. (2018). Fatigue, Not Self-Rated Motor Symptom Severity, Affects Quality of Life in Functional Motor Disorders. J. Neurol..

[56] Butler M., Shipston-Sharman O., Seynaeve M., Bao J., Pick S., Bradley-Westguard A., Ilola E., Mildon B., Golder D., Rucker J. (2021). International Online Survey of 1048 Individuals with Functional Neurological Disorder. Eur. J. Neurol..

