# Supplementary material for: Diagnostic Overshadowing in Functional Neurological Disorder Leading to a Diagnosis of Acute Motor and Sensory Axonal Neuropathy: A Case Report
Source: J Clin Med. 2026 May 3;15(9):3501. doi: 10.3390/jcm15093501 (PMC13163762; doi:10.3390/jcm15093501)

Supplemental File: Imaging Results

Waveforms:

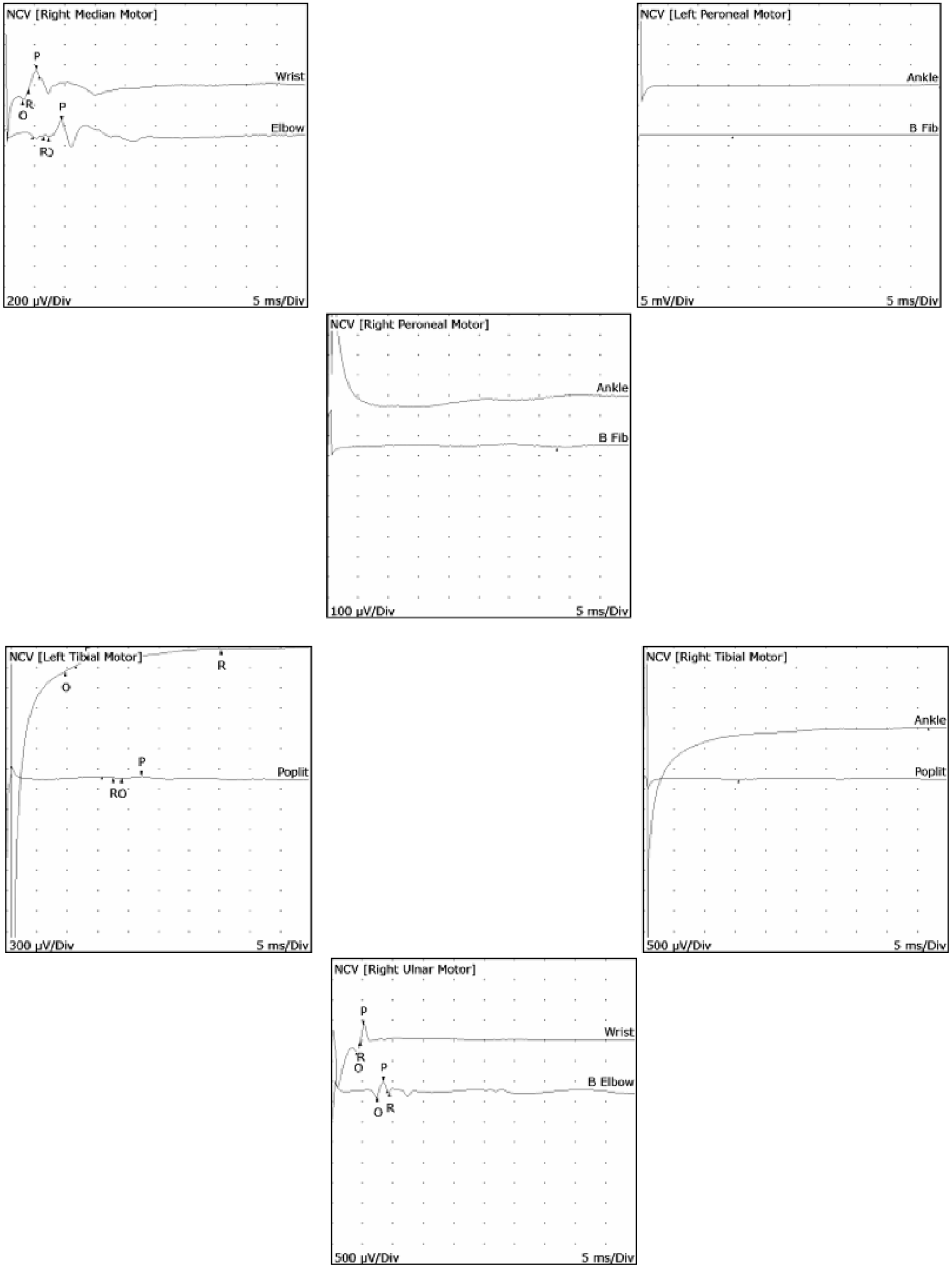

Supplemental File: Imaging Results

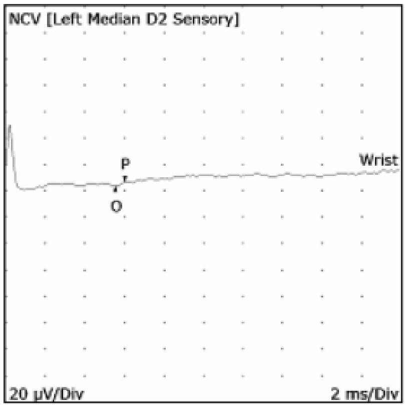

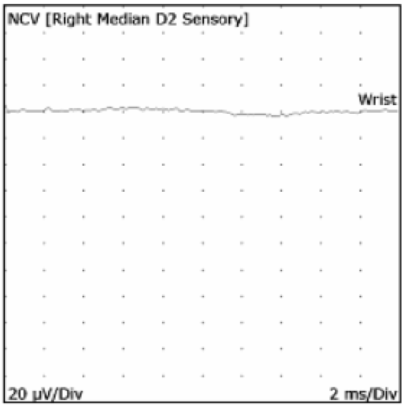

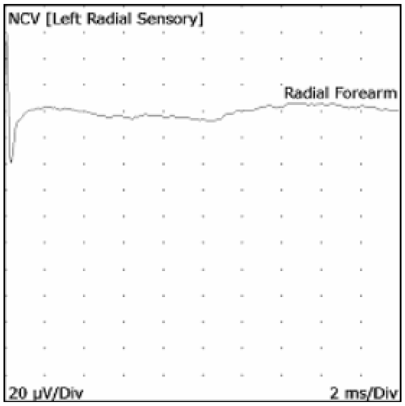

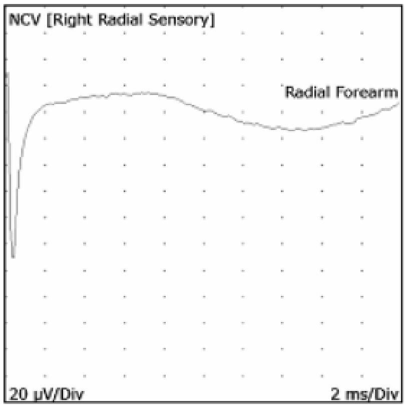

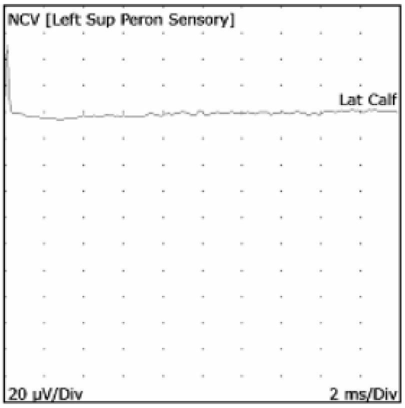

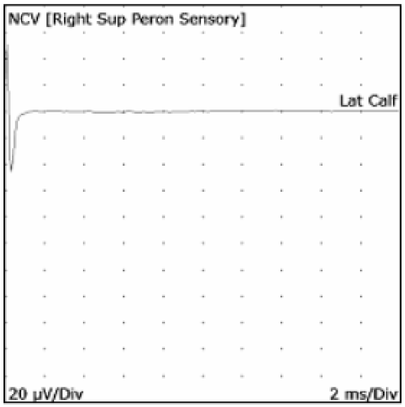

Supplemental File: Imaging Results

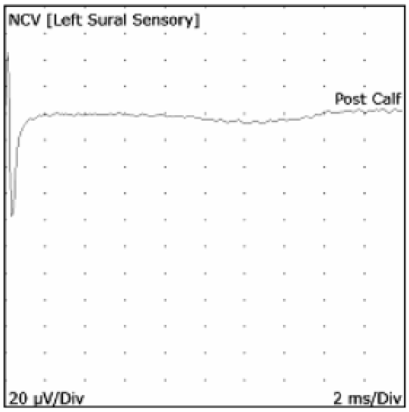

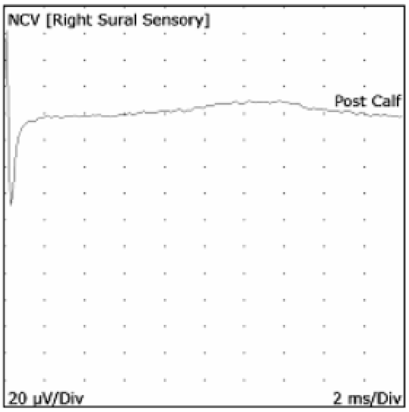

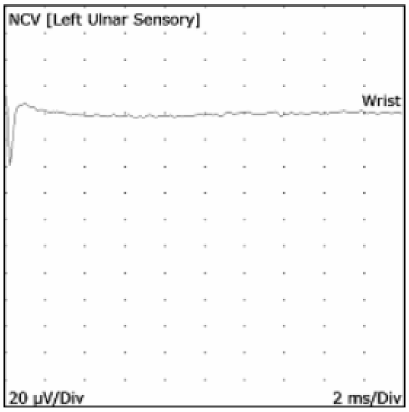

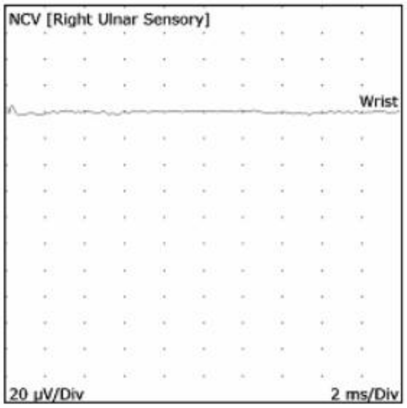

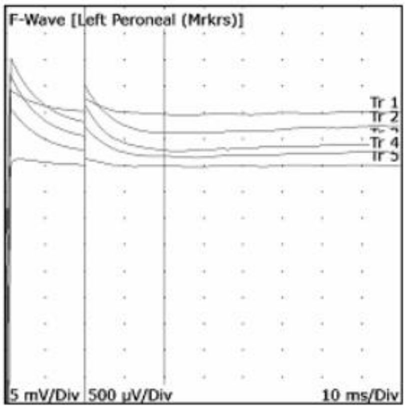

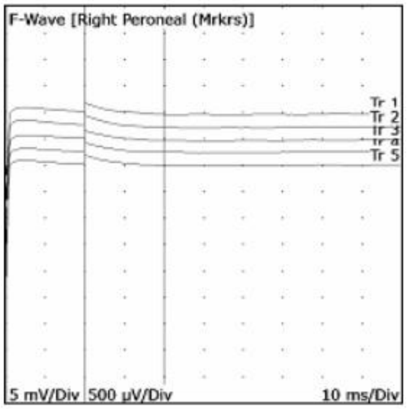

Supplemental File: Imaging Results

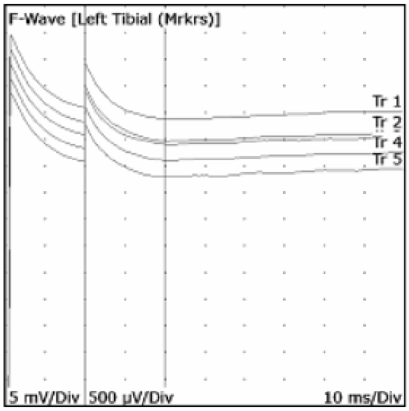

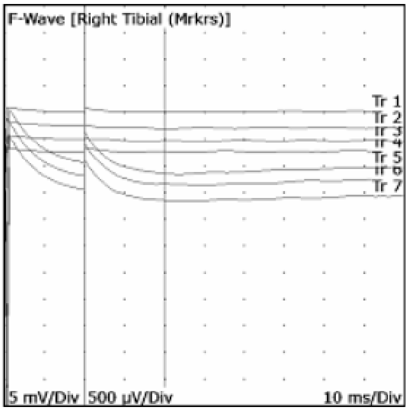

T2-weighted MRI

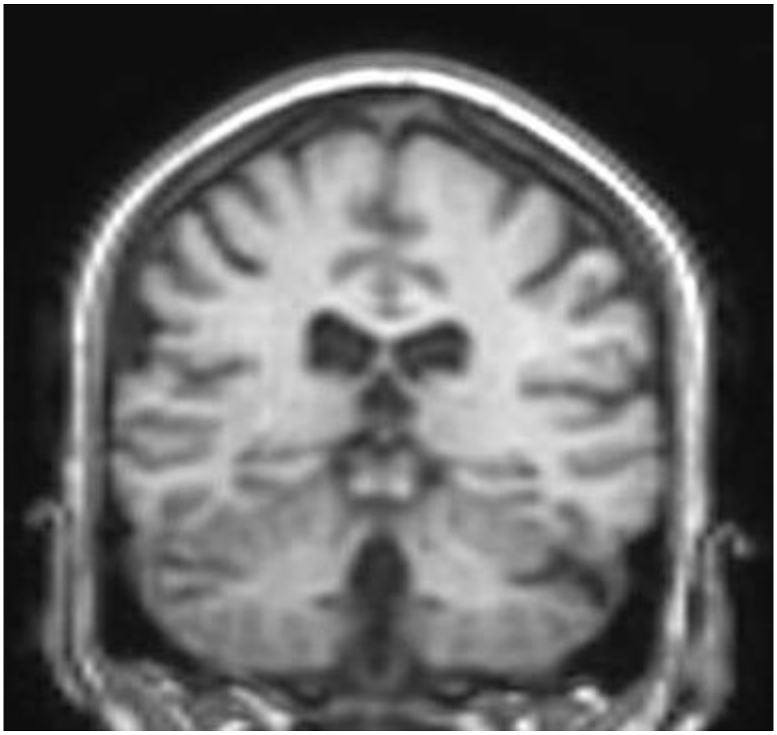

Supplement: Supplementary file 1 [file jcm-15-03501-s001.zip › jcm-4227991-supplementary.pdf]
